# Orthostatic hypotension and acute cognitive dysfunction in Parkinson’s disease: insights from functional near-infrared spectroscopy

**DOI:** 10.1016/j.clinph.2026.2112351

**Published:** 2026-07-27

**Authors:** Katherine Longardner, Pratusha Reddy, Danielle Shoshany, Michael Skipworth, Dawn M. Schiehser, David P. Salmon, Roy Freeman, Patricia A. Shewokis, Irene Litvan, Kurtulus Izzetoglu

**Affiliations:** a University of California San Diego, Department of Neurosciences, 9500 Gilman Dr. MC 0886 La Jolla, CA 92093, USA; b Drexel University, Department of Bioengineering, 3508 Market St, Room 102, Philadelphia, PA 19104, USA; c University of Florida, Norman Fixel Institute for Neurological Diseases, Department of Clinical and Health Psychology, P.O. Box 100165, Gainesville, FL 32610, USA; d Research Service, North Florida/South Georgia VA Healthcare System, 1601 SW Archer Rd, Gainesville, FL 32608, USA; e Department of Neurology, Beth Israel Deaconess Medical Center, Harvard Medical School, 330 Brookline Ave, Boston, MA 02215, USA

**Keywords:** Functional near-infrared spectroscopy, Parkinson’s disease, Orthostatic hypotension, Cognitive impairment, Blood pressure, Verbal fluency

## Abstract

**Objective:**

To examine short-term associations among position-dependent executive performance and cerebral hemodynamic changes using functional near-infrared spectroscopy (fNIRS) in participants with Parkinson’s disease (PD) with and without orthostatic hypotension (OH).

**Methods:**

Twenty-nine nondemented participants with PD (19 without OH (OH−), 10 with OH (OH+)) completed a verbal fluency test (VFT) in supine and upright positions on a tilt table, while their cerebral oxygenation was continuously monitored using a full-head fNIRS system. Age-adjusted ANCOVA examined the interaction effects of OH status and position on VFT scores, while linear mixed-effects models assessed changes in oxygenated (HbO) and deoxygenated hemoglobin (HbR) within the dorsolateral prefrontal cortex (DLPFC).

**Results:**

A significant interaction between OH status and position emerged for letter fluency (*p* = 0.043, η_p_^2^ = 0.148), with the OH+ group demonstrating poorer performance and more errors while upright. fNIRS analyses revealed that OH+ participants exhibited reduced HbR in both left (*p* = 0.010, *d* = 0.73, 95% CI: [0.19, 1.27]) and right DLPFC (*p* = 0.011, *d* = 0.73, 95% CI: [0.19, 1.27]) when upright, suggesting increased utilization of cognitive resources. No participants reported orthostatic symptoms during the cognitive task.

**Conclusions:**

Despite lack of OH symptomatology, PD OH+ individuals demonstrated impaired cerebral hemodynamic regulation when upright, which was associated with poorer cognitive performance.

**Significance:**

fNIRS-derived measures for monitoring cerebral hemodynamics in patients with autonomic failure may provide a clinically relevant indicator of postural changes in brain dysfunction.

## 1. Introduction

Parkinson’s disease (PD) is the second most common and fastest-growing neurodegenerative disorder worldwide (Dorsey et al. 2018). In addition to its hallmark motor symptoms (i.e., slowed movement, rest tremor, and muscular rigidity), PD frequently causes disabling non-motor symptoms, including cognitive impairment and autonomic nervous system dysfunction (Antonini et al. 2012). Neurogenic orthostatic hypotension (OH), a sustained fall in blood pressure (BP) when upright due to cardiovascular autonomic failure, affects about one-third of persons with PD (Wang et al. 2025). OH can lead to transient cerebral hypoperfusion and is associated with acute, reversible cognitive deficits when upright. Multiple studies have demonstrated that otherwise asymptomatic individuals with OH (OH+) perform worse on executive, memory, attention, and visuospatial tasks while upright compared to supine, whereas individuals without OH (OH−) do not show postural cognitive changes (Poda et al. 2012; Centi et al. 2017; Sforza et al. 2018). These temporary, positional cognitive impairments raise the possibility that OH-induced cerebral hypoperfusion may cumulatively contribute to longitudinal cognitive decline. Extending these observations, OH is strongly linked to chronic cognitive impairment across stages of PD. A systematic review and meta-analysis concluded that individuals with PD have 3.3-fold greater odds of either mild cognitive impairment or dementia if they have OH (Loureiro et al. 2023).

**Fig. 1.**
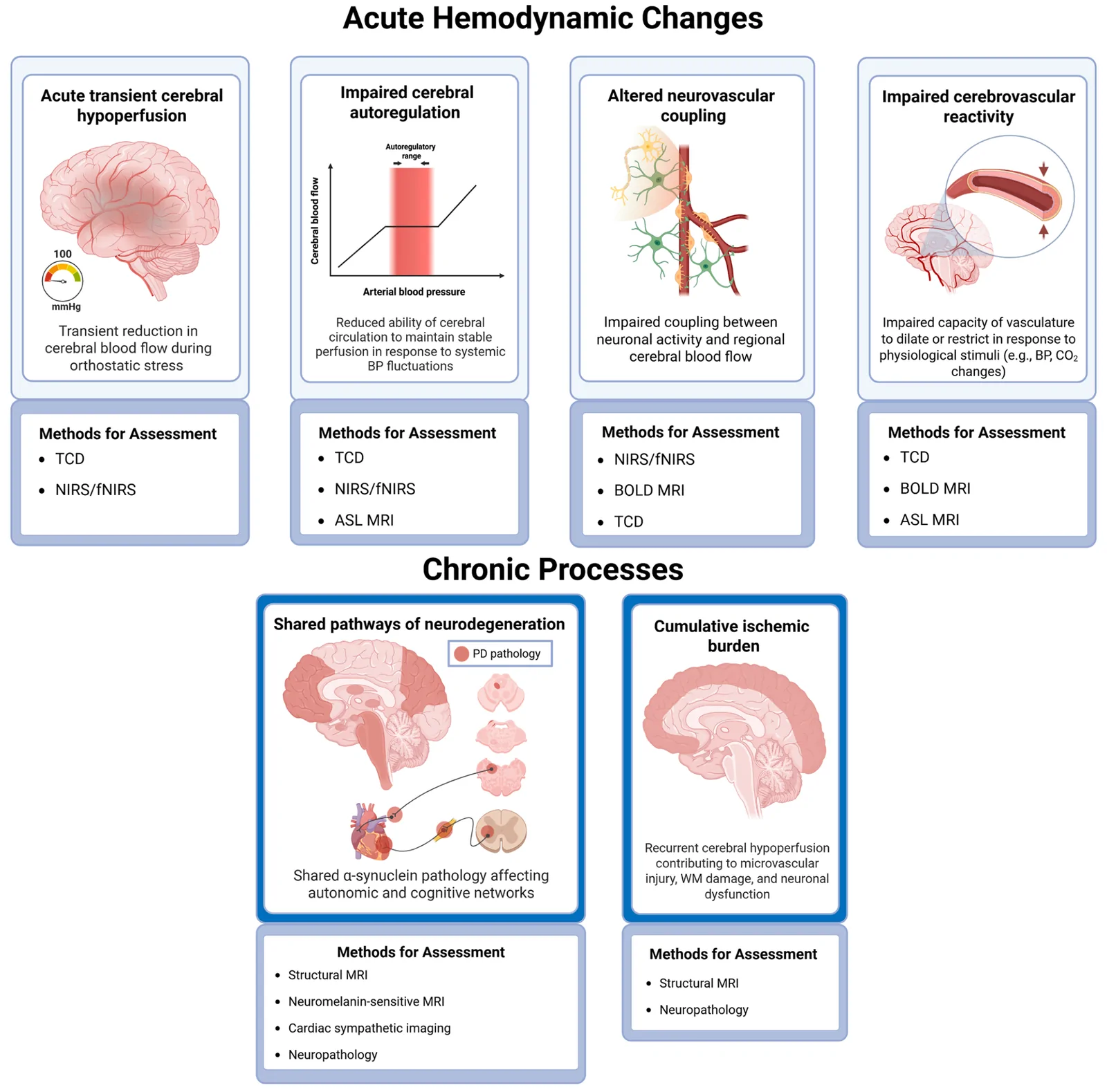
Schematic illustrating potential acute and chronic pathophysiological mechanisms linking orthostatic hypotension to cognitive impairment in Parkinson’s disease, with representative assessment modalities. Assessment of acute cerebral hypoperfusion and cerebral autoregulation requires concurrent cerebral hemodynamic and blood pressure monitoring during an orthostatic challenge, such as head-up tilt or, for MRI-based approaches, lower-body negative pressure. Assessment of neurovascular coupling requires a cognitive or sensory stimulus. Assessment of cerebrovascular reactivity requires a vasodilatory challenge. Abbreviations: ASL MRI: arterial spin labeling magnetic resonance imaging; BOLD: blood oxygenation level-dependent imaging; BP: blood pressure; NIRS: near-infrared spectroscopy; TCD: transcranial Doppler ultrasound; WM: white matter.

OH in PD may contribute to both acute and chronic cognitive impairment through a series of potentially interrelated mechanisms. Acutely, orthostatic stress can result in transient cerebral hypoperfusion, leading to reductions in cerebral blood flow and oxygen delivery. This effect may be exacerbated by impaired cerebral autoregulation, which compromises the normal ability to maintain stable perfusion despite fluctuations in systemic BP (Vokatch et al. 2007; Indelicato et al. 2015). Concurrently, alterations in neurovascular coupling may disrupt the appropriate increase in regional blood flow in response to neuronal activity, further limiting the brain’s capacity to meet metabolic demands during cognitive tasks (Azevedo et al. 2011; Shang et al. 2021). Additionally, impaired cerebrovascular reactivity may reduce vascular responsiveness to physiological stimuli such as changes in BP or carbon dioxide levels (Ryman et al. 2023). Over longer time scales (months to years), recurrent episodic hypoperfusion may lead to cumulative ischemic burden, promoting microvascular injury and white matter changes (Indelicato et al. 2015; Harmsen et al. 2018; Dadar et al. 2020), while diffuse PD-related neurodegeneration may simultaneously disrupt autonomic and cognitive networks (Fereshtehnejad et al. 2015; Horsager and Borghammer 2024). Together, these mechanisms provide a potentially synergistic framework through which cerebral hemodynamic dysfunction may contribute to cognitive impairment in PD (Udow et al. 2016; McDonald et al. 2016) (Fig. 1). Despite evidence linking OH to both short- and long-term cognitive impairment, the relationship between systemic BP fluctuations and cerebral hemodynamics in PD remains poorly defined, particularly under dynamic orthostatic conditions.

Progress in elucidating the relationship between OH, cognitive impairment, and cerebral hemodynamics has been limited by methodological challenges. Traditional neuroimaging techniques, such as functional magnetic resonance imaging (fMRI), cannot be performed in the upright position. Lower body negative pressure can induce a reduction in BP during supine neuroimaging (Kimmerly et al. 2005), but it remains uncertain whether this approach replicates the physiological changes that occur during real-world postural transitions. While transcranial Doppler (TCD) ultrasound can measure cerebral blood flow velocity in large intracranial arteries while upright, this method does not adequately characterize regional brain perfusion at the microvascular level. TCD studies of individuals with PD and related disorders have yielded inconsistent findings regarding cerebral autoregulation, ranging from normal (Niehaus et al., 2002; Angeli et al., 2003; Gurevich et al., 2006; Haubrich et al., 2010) to impaired (Vokatch et al., 2007; Camargo et al., 2015; Chen et al., 2022), with some suggesting an enhanced autoregulatory capacity that enables greater tolerance to BP fluctuations (Novak et al., 1998).

To overcome limitations of existing modalities in monitoring posture-dependent cerebral hemodynamic changes during cognitive tasks, we employed a portable functional near-infrared spectroscopy (fNIRS) system. This non-invasive, wearable neuroimaging technique applies light at distinct wavelengths to the scalp to measure variations in two chromophores in the blood: oxygenated (HbO) and deoxygenated (HbR) hemoglobin. Neuronal activation in response to a cognitive task increases brain tissue’s metabolic demand, which increases regional cerebral perfusion, leading to increased HbO and decreased HbR relative to baseline (i.e., hemodynamic responses to stimuli) (Huppert et al., 2006). Thus, fNIRS is an ideal candidate to assess position-induced cerebral hemodynamic changes since it can be used while participants are upright or supine and is capable of continuously detecting real-time regional brain activity in the superficial cortex with demonstrated validity and reliability (Udina et al. 2019; Pinti et al. 2020). While other techniques for monitoring cerebral hemodynamics provide complementary insights, fNIRS uniquely enables real-time assessment of cortical hemodynamics and neurovascular coupling during orthostatic stress (Supplementary Table 1).

Expanding on earlier work that examined acute OH-related cognitive changes in persons with PD (Centi et al., 2017; Poda et al., 2012; Sforza et al., 2018), the present prospective, cross-sectional observational study aimed to determine how fNIRS biomarkers relate to executive performance while participants were supine and upright. We focused on executive performance because executive dysfunction is the most common PD-related cognitive disturbance and compared position-dependent changes between participants with PD with and without OH. We hypothesized that the OH+ group would demonstrate poorer cognitive performance during upright testing as compared to supine testing. Accordingly, we anticipated a greater increase in HbO and a greater reduction in HbR during upright testing compared to supine testing, indicating a greater use of cognitive resources in the upright position. In contrast, the OH− group was not expected to exhibit positional differences in cognitive performance or HbO/HbR.

## 2. Materials and methods

### 2.1. Study design

The study protocol was approved by the University of California San Diego (UCSD) Institutional Review Board (Project # 210738) and registered on ClinicalTrials.gov (Identifier: NCT05400174). All participants provided written informed consent prior to enrollment. Participants were recruited from local movement disorders clinics, PD support groups, and community outreach events. Enrollment took place between December 14, 2021, and January 24, 2025. All study procedures were conducted at UCSD. Study data were collected and managed using Research Electronic Data Capture (REDCap) tools hosted at UCSD (Harris et al. 2009, Harris et al., 2019). The REDCap randomization tool was used as described below.

### 2.2. Study population

Eligibility was confirmed by a movement disorders neurologist (K. L.). Enrolled participants met the following inclusion criteria: (1) diagnosis of idiopathic PD using the Movement Disorder Society (MDS) Clinical Diagnostic Criteria (Postuma et al. 2015); (2) age at least 50 years old; (3) Hoehn & Yahr stage III or lower (early to moderate stage PD; able to walk without assistance from another person) (Hoehn and Yahr 1967); and (4) proficiency in the English language. Exclusion criteria were: (1) frequent involuntary movements (e.g., tremor or dyskinesia) with amplitude greater than 3 cm since motion artifact can interfere with BP measurements; (2) dementia, including PD dementia (Emre et al. 2007), characterized by either a Mattis Dementia Rating Scale-2 (DRS-2) (Jurica et al. 1988) score of 132 or less (Matteau et al. 2012; Turner and Hinson 2013) or clinical evidence of impaired instrumental activities of daily living due to cognitive impairment; (3) status post deep brain stimulation surgery; (4) any unstable, active medical problem, such as decompensated heart failure, acute pneumonia, etc.; (5) moderate or severe carotid artery stenosis according to North American Symptomatic Carotid Endarterectomy Trial criteria (North American Symptomatic Carotid Endarterectomy Trial Steering Committee, 1991); (6) history of cerebral infarction or hemorrhage since this could affect fNIRS signals and/or cognitive performance; (7) uncontrolled diabetes mellitus or any other systemic disease causing autonomic failure; (8) taking BP-lowering medications, including anti-hypertensives or α-adrenoreceptor antagonists; (9) any terminal illness with a life expectancy of less than two years; (10) illiteracy; (11) impairment of hearing or vision not corrected by adaptive devices (e.g., hearing aids or glasses); (12) currently pregnant; or (13) any other condition that could place the participant at increased risk, including syncope within the past week.

Participants attended a screening visit at which they provided demographic information and medical history and underwent a physical and neurological examination, which included the Movement Disorder Society Unified PD Rating Scale (MDS-UPDRS) Part III to assess motor performance (Goetz et al. 2008) and the Hoehn & Yahr scale (Hoehn and Yahr 1967). PD duration was determined based on the date of reported motor symptom onset. Levodopa equivalent daily dose (LEDD) was calculated using previously described methodology (Tomlinson et al. 2010). Global cognitive function was assessed while participants were seated using the DRS-2 (Jurica et al. 1988). The DRS-2 evaluates overall cognition across multiple domains: initiation/perseveration (executive function), construction (visuospatial function), conceptualization (executive function), attention, and memory. The DRS-2 was selected as a screening instrument because it is one of three cognitive assessment tools recommended without caveats by the MDS for evaluating global cognitive performance in PD (Skorvanek et al. 2018). It demonstrates strong reliability and validity, is sensitive to longitudinal change, has established clinical utility in distinguishing PD dementia from mild cognitive impairment (Matteau et al. 2011), and is associated with functional impairment in activities of daily living (Rosenthal et al. 2010). Furthermore, it evaluates the cognitive domains necessary to diagnose PD-mild cognitive impairment, consistent with MDS recommendations (Litvan et al. 2011, Litvan et al., 2012).

During the screening visit, participants underwent a head-up tilt table test using a motorized bed (Balance Tuscan Tilt Table, Stonehaven Medical, Inc.). BP and heart rate (HR) were measured after five minutes of supine rest. The tilt table was then raised to an angle of 70° and maintained upright for three minutes, with BP and HR measured at one and three minutes after the tilt. OH was defined as a systolic BP (SBP) drop of at least 20 mmHg and/or a diastolic BP (DBP) drop of at least 10 mmHg sustained at three minutes of head-up tilt, according to consensus criteria (Gibbons et al. 2017). Participants were initially classified as OH+ or OH− at the screening visit based on BP assessment performed before 1:1 randomization to testing order. Randomization determined only whether participants completed the upright or supine condition first and was independent of OH status. OH status was then reassessed during the study visit and OH classification at that visit was used for the primary analyses.

#### 2.2.1. Study visit procedures

Participants attended a study visit scheduled as soon as feasible following the screening visit (mean interval between the screening visit and the study visit: 41.4 days, SD = 26.1). Study visits were scheduled within the three-hour period between 9:00 a.m. and 12:00 p.m. to minimize diurnal BP fluctuations. Participants were instructed to abstain from caffeine for 24 h before the visit, to eat a small meal beforehand, and to continue all routine medications, including antihypotensive and antiparkinsonian agents, as applicable. Participants taking levodopa were instructed to arrive in the “on” state.

#### 2.2.2. Cognitive testing

As part of a larger cognitive battery administered during alternating supine and upright tilt-table testing following previously described methods (Centi et al. 2017), participants completed the letter fluency condition of the Delis-Kaplan Executive Function System (D-KEFS) Verbal Fluency Test (VFT) (Delis et al. 2012). The broader battery assessed multiple cognitive domains, including memory, visuospatial function, and attention, and was divided into four approximately 20-minute testing segments with alternating positions (e.g., supine–upright–supine–upright or the reverse). The D-KEFS VFT was administered at the beginning of the second and third testing segments, resulting in an interval of approximately 20 minutes between administrations. During this letter fluency task, individuals generate as many words as possible beginning with a specific letter of the alphabet within one minute, then repeat the process using three different letters in total (F-A-S or B-H-R). To minimize practice effects, alternate test versions were administered in the supine and upright positions. Version order (F-A-S or B-H-R) was randomized 1:1. Errors were categorized according to standard scoring guidelines as follows: (1) perseverations, defined as repetitions of previously generated words; (2) intrusions, which included words that did not begin with the target letter or otherwise violated task rules; and (3) set-loss errors, defined as losing the task set by briefly shifting to an inappropriate retrieval strategy (e.g., generating semantically related items rather than letter-based responses). The primary cognitive outcome was the total number of correct words generated. The total number of errors was chosen as a secondary outcome.

The D-KEFS was selected because it provides a standardized and well-validated framework that encompasses multiple fluency conditions (letter fluency, category fluency, and category switching fluency). In this study, analyses focused on the letter fluency condition given that it reflects executive dysfunction. The D-KEFS also provides detailed performance metrics – including total correct responses, total incorrect responses, error subtypes (e.g., perseverations, intrusions, and set-loss errors), and total responses – that allow for more granular characterization of cognitive performance. Verbal letter fluency was selected as the primary outcome for the present study because it primarily measures executive function, the cognitive domain most affected in PD (Pettit et al. 2013). Furthermore, impairment in letter fluency predicts progression to PD dementia (Jacobs et al. 1995). The D-KEFS letter fluency test, in particular, serves as an early, sensitive marker of PD-related cognitive decline (Petkus et al. 2024). Additionally, as a measure of executive function, it is well suited to our experimental paradigm because it requires no drawing or manual manipulation, thereby minimizing confounding by PD-related motor impairment while allowing standardized testing in both the supine and upright positions.

#### 2.2.3. Tilt table testing and vital sign measurements

During upright testing, the tilt table was raised to an angle of 70° and remained tilted at that angle at rest for three minutes before beginning the VFT. A brachial oscillometric sphygmomanometer (ADC^®^; ADView^®^ 2, American Diagnostic Corporation, Hauppauge, NY) was used to record BP and HR every two minutes throughout the study visit. Oscillometric BP monitoring was not performed in the first five participants due to iterative refinements in the protocol during the initial phase of the study. All subsequent participants underwent BP measurements using the finalized protocol.

#### 2.2.4. Orthostatic symptom assessment

We evaluated all participants for orthostatic symptoms over the preceding week and during cognitive testing. At the beginning of the study visit, participants completed the Orthostatic Hypotension Questionnaire (OHQ), which assesses the severity of and disability from OH symptoms within the past week (Kaufmann et al. 2012). The Orthostatic Hypotension Symptom Assessment (OHSA) composite score was calculated as previously described (Kaufmann et al. 2025) by averaging the scores of the first six questions of the OHQ, which assess the following symptoms of organ hypoperfusion while upright: (1) dizziness, lightheadedness, and/or near-fainting, (2) visual disturbances, (3) difficulty concentrating, (4) weakness, (5) fatigue, and (6) coat-hanger pain. During the cognitive testing, participants were instructed to report any orthostatic symptoms (e.g., lightheadedness, dizziness, or feeling faint) experienced while upright. Upright testing was halted if participants reported any orthostatic symptoms severe enough to interfere with task completion or showed signs of impending syncope (e.g., decreased responsiveness). At the end of each upright testing period, participants were queried about any orthostatic symptoms experienced during testing, and these were recorded.

#### 2.2.5. fNIRS data acquisition

Cerebral hemodynamic changes were monitored using a continuous-wave fNIRS system (NIRSport2, NIRx^®^, Berlin, Germany). The sensor array included 16 surface-mount light-emitting diodes (LEDs) and 23 silicon photodiodes. The montage is illustrated in Supplementary Fig. S1. With this source-detector configuration, the system collected data from 44 long source-detector separation (SDS) and 8 short SDS measurements across the whole head. Long SDS data from eight channels located over the prefrontal cortex were used for the purposes of the present study (see highlighted channels in Supplementary Fig. S1) given this cortical area’s role in letter fluency (Tupak et al. 2012). To minimize ambient light artifacts, an opaque cap was placed over the fNIRS setup. When not engaged in cognitive tasks, participants were instructed to rest quietly with their eyes open. Event markers were used to indicate transitions between supine and upright positions and the start and end of each cognitive task.

### 2.3. fNIRS data processing

MATLAB (MathWorks, R2022a) was used to preprocess light intensity data and calculate fNIRS measures. The processing pipeline within the light-intensity domain consisted of removing channels that were saturated or had low light intensity values, followed by detrending a third-order polynomial fit to remove abrupt spikes and removing motion artifacts via wavelet-based motion artifact removal (Sweeney et al. 2011; Molavi and Dumont 2012; Reddy et al. 2021). The modified Beer-Lambert law was then used to calculate changes in the concentration of HbO and HbR, using age- and wavelength-corrected differential pathlength factor (Villringer and Chance 1997; Scholkmann and Wolf 2013). Low-frequency drifts and high-frequency noise associated with respiration and cardiac activity were removed via high- and low- pass finite impulse response filters with cutoff frequencies at 0.005 and 0.1 Hz (Reddy et al. 2021; Izzetoglu et al. 2007). Time-series HbO and HbR data across channels were averaged to obtain mean changes in the left and right dorsolateral prefrontal cortex (DLPFC) given this region’s established role in letter fluency tasks (Herrmann et al. 2003; Huang et al. 2016).

### 2.4. Statistical analysis

Demographic and clinical characteristics were summarized by OH status (OH− vs. OH+) during the study visit. Continuous variables were compared between groups using independent-samples t-tests when distributional assumptions were reasonable; categorical variables were compared using chi-square tests or Fisher’s exact tests when expected cell counts were small. Assumption checks, including assessment of normality, homogeneity of variance, residual distributions, and influential observations, were conducted before inferential analyses and are reported in the Supplementary Material. For VFT outcomes, two-way mixed ANCOVA models were used, with OH group as the between-subjects factor, position as the within-subjects factor, and age as a covariate. Age was selected a priori because it differed between OH groups and is a known determinant of cognitive performance. Given the relatively small sample size and imbalance between OH groups, covariate adjustment in the primary models was intentionally limited to age to reduce the risk of overfitting and unstable parameter estimates, particularly in the smaller OH+ subgroup. Other clinically relevant variables, including sex, levodopa equivalent daily dose (LEDD), and antihypotensive medication use, were summarized descriptively. Where feasible, sensitivity analyses were planned by adding these covariates individually to the primary model to determine whether the direction or interpretation of the OH group × position effect changed materially.

Because VFT error scores were count-based, sparse, and skewed, the ANCOVA assumptions were examined carefully for this outcome. As a sensitivity analysis, VFT error counts were also analyzed using a generalized mixed-effects model appropriate for count data, with fixed effects for OH group, position, and the OH group × position interaction, and a participant-level random intercept to account for repeated measurements. A Poisson model was considered initially, with overdispersion evaluated; if overdispersion was evident, a negative binomial specification was preferred. These analyses were used to assess whether conclusions regarding the OH group × position effect were consistent with those from the ANCOVA. Because error counts were sparse, findings from these models were interpreted cautiously and treated as supportive rather than primary evidence.

Linear mixed-effects regression models were used to analyze fNIRS and physiologic outcomes because observations were repeated within participants and because this modeling approach permits inclusion of all available observations under a missing at random assumption. Patterns of missingness were examined, and no imputation was performed. The number of available observations in each OH group × position cell is reported in the Supplementary Material.

Separate linear mixed-effects models were fit for each fNIRS outcome and each physiologic outcome. The fNIRS dependent variables were mean HbO and HbR in the left and right DLPFC. Physiologic dependent variables included systolic BP, diastolic BP, mean arterial pressure (MAP), and HR. For each dependent variable, the primary model included fixed effects for OH group, position, and the OH group × position interaction, with a random intercept for participant in Equation (1):

(1) DVij=β0+β1(OH Groupi)+β2(Positionij)+β3(OH Groupi×Positionij)+b0i+∈ij

b0i represents the participant-specific random intercept, and ∈ij represents the residual error. The primary test of interest for each model was the OH Group ×Position interaction.

In R notation, the model was specified as:

DV~Group*Position+(1|ID).

Fixed effects were evaluated using likelihood ratio tests comparing the full model with a reduced model omitting the effect of interest. For the interaction test, the full model was compared with a reduced model containing the OH group and position main effects but omitting the OH group × position interaction. Maximum likelihood estimation was used for likelihood ratio testing, whereas restricted maximum likelihood estimation was used to estimate final model parameters and post hoc contrasts. When a significant OH group × position interaction was observed, planned contrasts were conducted to compare OH groups within each position and positions within each OH group. The Satterthwaite approximation was used to estimate degrees of freedom for post hoc tests (Satterthwaite, 1941, 1946).

Model assumptions for the linear mixed-effects models were evaluated using visual inspection of residual plots, fitted-versus-residual plots, and random-effect distributions. When residual non-normality or heteroscedasticity was evident, a log10 transformation of the dependent variable was considered. When residual non-normality or heteroscedasticity was evident, alternative specifications or transformations appropriate to the dependent variable's scale and distribution were considered. Transformations were not applied when they would compromise interpretability or were inappropriate for variables containing zero or negative values. Results from transformed models were used when they improved model diagnostics without altering interpretability.

For fNIRS analyses, planned post hoc contrasts were treated as a comparison family separately for each dependent variable: left DLPFC HbO, right DLPFC HbO, left DLPFC HbR, and right DLPFC HbR. To limit Type I error inflation, false discovery rate correction was applied within each family of planned post hoc comparisons. Both unadjusted and false discovery rate–adjusted p values are reported in the Supplementary Tables S6–S8. The same approach was used for physiologic outcomes when multiple planned comparisons were conducted within a dependent variable. Effect sizes for post hoc comparisons were summarized using Cohen’s d, with small, medium, and large effect sizes defined as approximately 0.20, 0.50, and 0.80, respectively (Cohen, 2013). All tests were two-sided, and statistical significance was defined as α = 0.05. ANCOVA and descriptive analyses were conducted using IBM SPSS Statistics for Windows, Version 29.0.2.0. Linear mixed-effects models for fNIRS and physiological variables were conducted in R using the *lme4*, *lmerTest*, and emmeans packages (Bates et al. 2015; Kuznetsova et al. 2017; Lenth 2023). Generalized mixed-effects models were fit using *glmmTMB* (Brooks et al., 2017), and model diagnostics were evaluated using simulation-based residuals from *DHARMa* (Hartig, 2026).

Sensitivity analyses were conducted to evaluate the robustness of the physiologic OH group × position interaction estimates to missing observations and potential confounding. First, the primary linear mixed-effects models, which used all available observations under a missing-at-random assumption, were compared with complete-case models restricted to participants with both supine and upright measurements. Second, age, sex, LEDD, and antihypotensive medication use were added individually to the primary model. A fully adjusted model containing all four covariates was also examined as an exploratory sensitivity analysis. Because of the modest and unbalanced sample size, these covariate-adjusted models were used to assess robustness rather than to provide definitive causal adjustment.

## 3. Results

### 3.1. Demographic and clinical characteristics

**Fig. 2.**
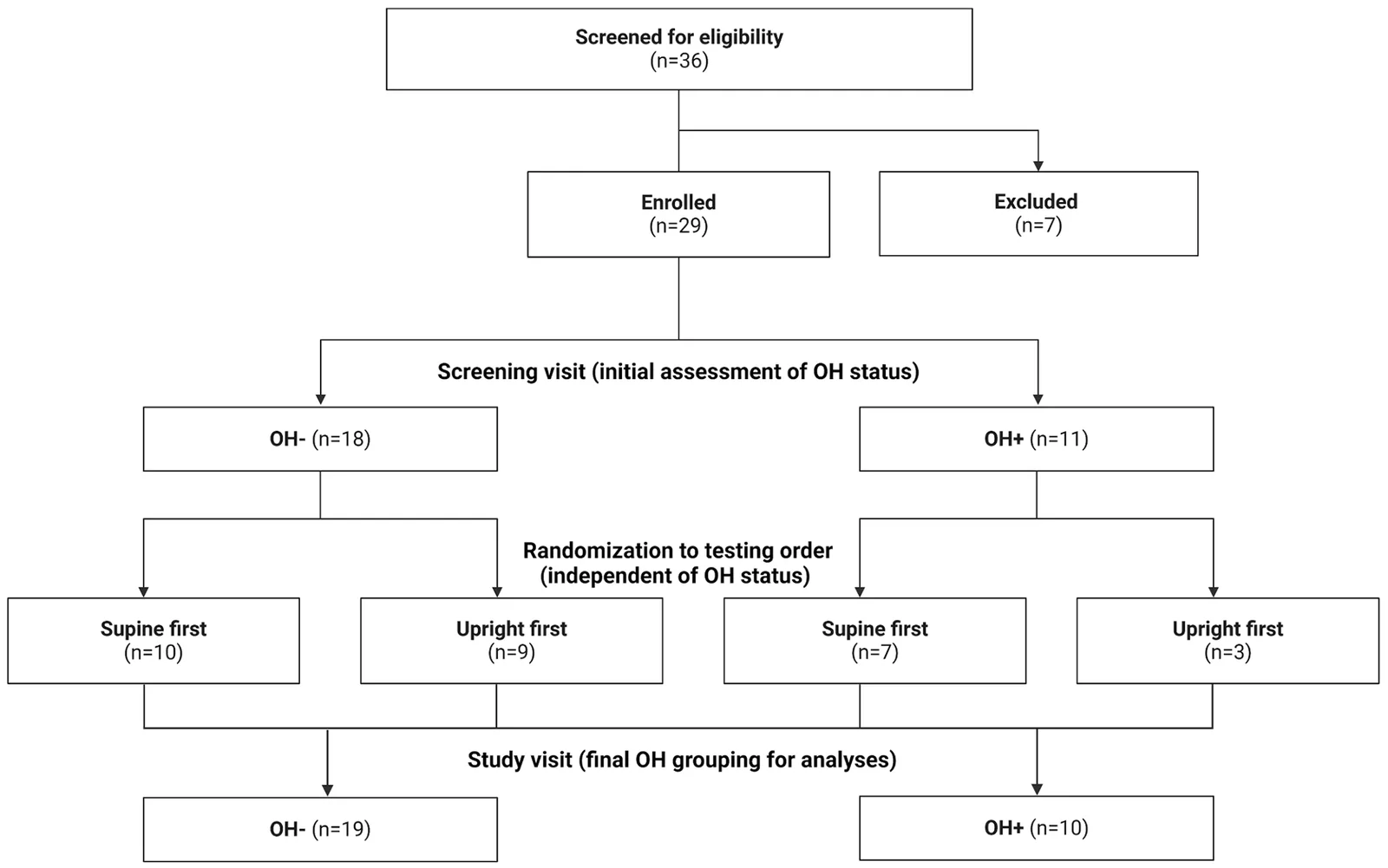
Flow diagram of participant screening, randomization, and study procedures. Participants underwent an initial orthostatic blood pressure assessment at the screening visit to provisionally classify orthostatic hypotension (OH) status before randomization. The starting posture (supine vs. upright) and test order (standard vs. alternate) were each randomized in a 1:1 ratio. Orthostatic blood pressure was reassessed during the study visit, and the presence or absence of OH at the study visit was used for the final analytic grouping.

Thirty-six participants were recruited, of whom 29 were eligible and enrolled (n = 19 OH−, n = 10 OH+). One participant met criteria for OH at screening but not during the study visit and was therefore classified as OH− for the primary analyses based on study visit physiology. All remaining participants had concordant OH classifications at the screening and study visits. The flow diagram of enrolled participants is shown in Fig. 2. Reasons for exclusion were contraindicated medications (n = 3), dementia (n = 2), recent syncope (n = 1), and a history of intracranial hemorrhage (n = 1). Table 1 shows clinical and demographic data from the enrolled participants and orthostatic vital signs measured during the screening visit tilt. The mean age of all participants was 67.3 years (standard deviation (SD) = 6.3), with the OH+ group being older (M = 70.8 years, SD = 4.5) than the OH− group (M = 64.9 years, SD = 6.1, *p* = 0.008, 95% confidence interval (CI): 1.82, 10.77). While 48.3% of all enrolled participants were female, 10% of the OH+ group and 68.4% of the OH− group were female, Х^2^(1) = 8.96, *p* = 0.003. Levodopa equivalent daily dose (LEDD) was greater in the OH+ group (M = 744.5 mg, SD = 416.5) than in the OH− group (M = 394.2 mg, SD = 208.6, *p* = 0.029, 95% CI: 43.38, 657.34). All ten OH+ participants were taking oral carbidopa/levodopa, and one was also taking rasagiline. In the OH− group, 17 participants (89.5%) were taking oral carbidopa/levodopa. Adjunctive therapies in the OH− group included rasagiline (n = 2), rotigotine (n = 1), and extended-release amantadine (n = 1), while two participants were treatment-naïve. Antihypotensive medication use was more common in the OH+ group: eight participants (80%) were taking at least one antihypotensive medication, including three who were taking multiple agents. Specific medications included fludrocortisone (n = 5), midodrine (n = 4), droxidopa (n = 4), and pyridostigmine (n = 1). In contrast, only one OH− participant (5.3%) was taking an antihypotensive medication (fludrocortisone), Х^2^(1) = 16.33, *p* < 0.001. OHQ item 1 scores (feeling dizzy, lightheaded, or faint when standing within the past week) were higher in the OH+ group (M = 3.1, SD = 2.8) compared to the OH− group (M = 0.90, SD = 2.0, *p* = 0.020, 95% CI: 0.38, 4.04). The OH− and OH+ groups were similar in education, PD duration, motor severity in the “on” state, global cognition, and OHSA composite scores.

### 3.2. Executive performance

**Fig. 3.**
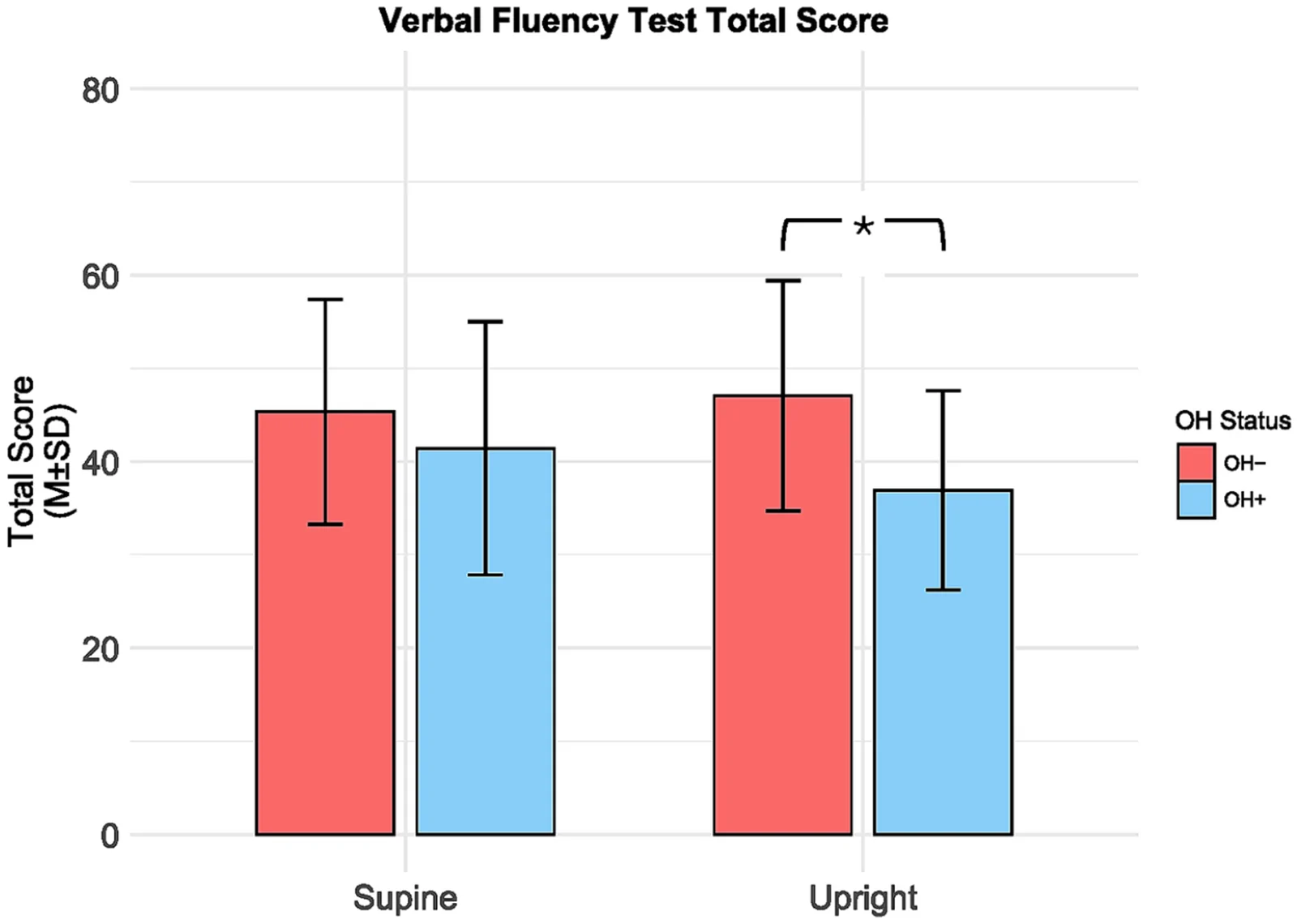
Unadjusted Delis-Kaplan Executive Function System letter verbal fluency test total scores in Parkinson’s disease participants by orthostatic hypotension (OH) status and position. Bars represent means ± SD. The asterisk indicates a significant unadjusted comparison between the OH− and OH+ groups in the upright position [*p* = 0.037]. In the unadjusted two-way mixed ANOVA, a between-group simple-effects contrast was used to test the effect of OH status within the upright position. Upright letter VFT total scores were significantly higher in the OH− group than in the OH+ group (47.05 ± 2.71 vs 36.90 ± 3.74 correct words), F(1, 27) = 4.834, p = 0.037, partial η^2^ = 0.152. Inferential conclusions were based on the age-adjusted mixed ANCOVA; the adjusted OH− versus OH+ comparison in the upright position was not significant [*p* = 0.131], although the OH status × position interaction was significant [*p* = 0.043]. Abbreviations: M, mean; SD, standard deviation; OH, orthostatic hypotension; OH−, without OH; OH+, with OH.

**Fig. 4.**
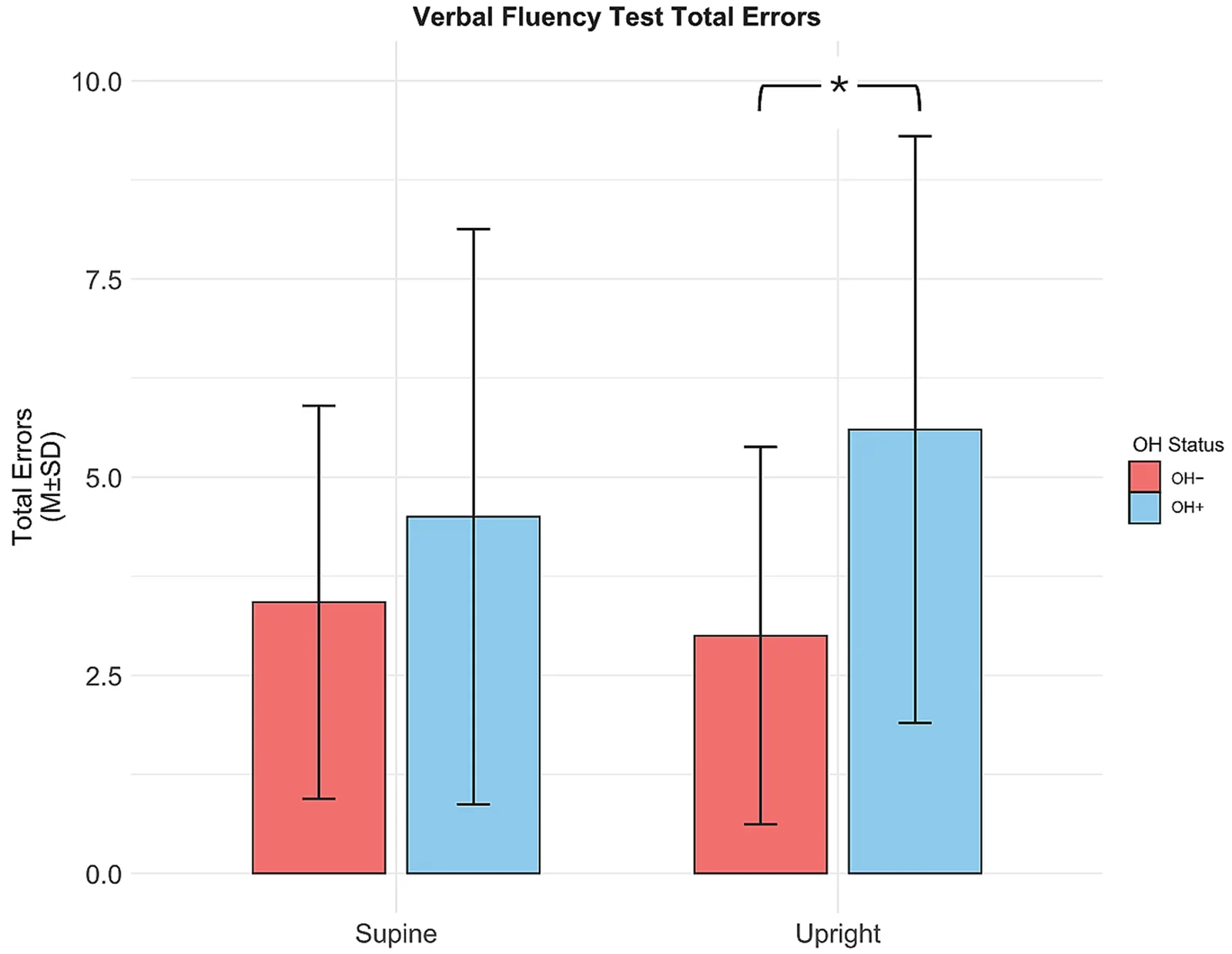
Delis-Kaplan Executive Function System letter verbal fluency test total errors in Parkinson’s disease participants without orthostatic hypotension (OH−) and with orthostatic hypotension (OH+) across supine and upright positions, controlling for age. Bars indicate group means; error bars represent 95% confidence intervals. Covariates are evaluated at age = 67.28. Significance: * p < 0.05. Abbreviations: M, mean; SD, standard deviation; OH, orthostatic hypotension; OH−, without OH; OH+, with OH.

Average letter VFT total and error scores for each group are illustrated in Fig. 3 and Fig. 4, respectively. The assumption of homogeneity of regression slopes was met for both dependent variables (the letter VFT score and total errors). The normality of residuals assumption was met for the letter VFT score, but not for total errors; this violation is expected, given that error counts are discrete and skewed (Poisson-like). The assumption of homogeneity of variance was fully met for both outcome measures. The linear specification for the age covariate was appropriate.

A two-way mixed ANCOVA, with position as the within-subject factor, OH status as the between-subject factor, and age as a covariate, identified a significant OH status × position interaction for letter VFT total score, *F*(1, 26) = 4.51, *p* = 0.043, partial η^2^ = 0.148 (Fig. 3). Fig. 3 presents the unadjusted scores, which were higher in the OH− than in the OH+ group in the upright position (47.05 ± 2.71 vs 36.90 ± 3.74 correct words; unadjusted comparison, p < .05). Adjusted marginal means were estimated at the sample mean age of 67.28 years and are reported as mean ± standard error (SE). After adjustment for age, the upright position difference was attenuated and was not statistically significant, although the adjusted letter VFT scores remained lower in the OH+ group than in the OH− group (38.11 ± 4.14 vs 46.41 ± 2.88 correct words), *F*(1, 26) = 2.43, *p* = 0.131, partial η^2^ = 0.085). In the supine position, adjusted scores were also similar between the OH+ and OH− groups (42.80 ± 4.40 vs 44.58 ± 3.06 correct words), *F*(1, 26) = 0.10, *p* = 0.756, partial η^2^ = 0.004). Within-group comparisons showed no significant positional difference in either the OH− group (upright minus supine: 1.84 ± 1.66 correct words), *F*(1, 26) = 1.22, *p* = 0.279, partial η^2^ = 0.045, or the OH+ group (−4.69 ± 2.38 correct words), *F*(1, 26) = 3.87, *p* = 0.060, partial η^2^ = 0.129. The age-adjusted main effect of OH status was not significant, *F*(1, 26) = 0.91, *p* = 0.349, partial η^2^ = 0.034.

For letter VFT total errors, the OH status × position interaction approached significance, *F*(1, 26) = 3.93, *p* = 0.058, partial η^2^ = 0.131 (Fig. 4). Exploratory age-adjusted analyses indicated that, in the upright position, the OH+ group made more errors (5.59 ± 0.94) than the OH− group (3.00 ± 0.66), *F*(1, 26) = 4.53, *p* = 0.043, partial η^2^ = 0.148. Error counts were similar between the OH+ (4.11 ± 1.01) and OH− (3.63 ± 0.70) groups in the supine position, *F*(1, 26) = 0.14, *p* = 0.712, partial η^2^ = 0.005. Within-group positional differences were not significant in either the OH− group (upright minus supine: −0.62 ± 0.57 errors), *F*(1, 26) = 1.18, *p* = 0.288, partial η^2^ = 0.043, or the OH+ group (1.48 ± 0.82), *F*(1, 26) = 3.24, *p* = 0.084, partial η^2^ = 0.111. The age-adjusted main effect of OH status on total errors was not significant, *F*(1, 26) = 1.81, p = 0.191, partial η^2^ = 0.065.

### 3.3. Changes in HbO and HbR during letter VFT

**Fig. 5.**
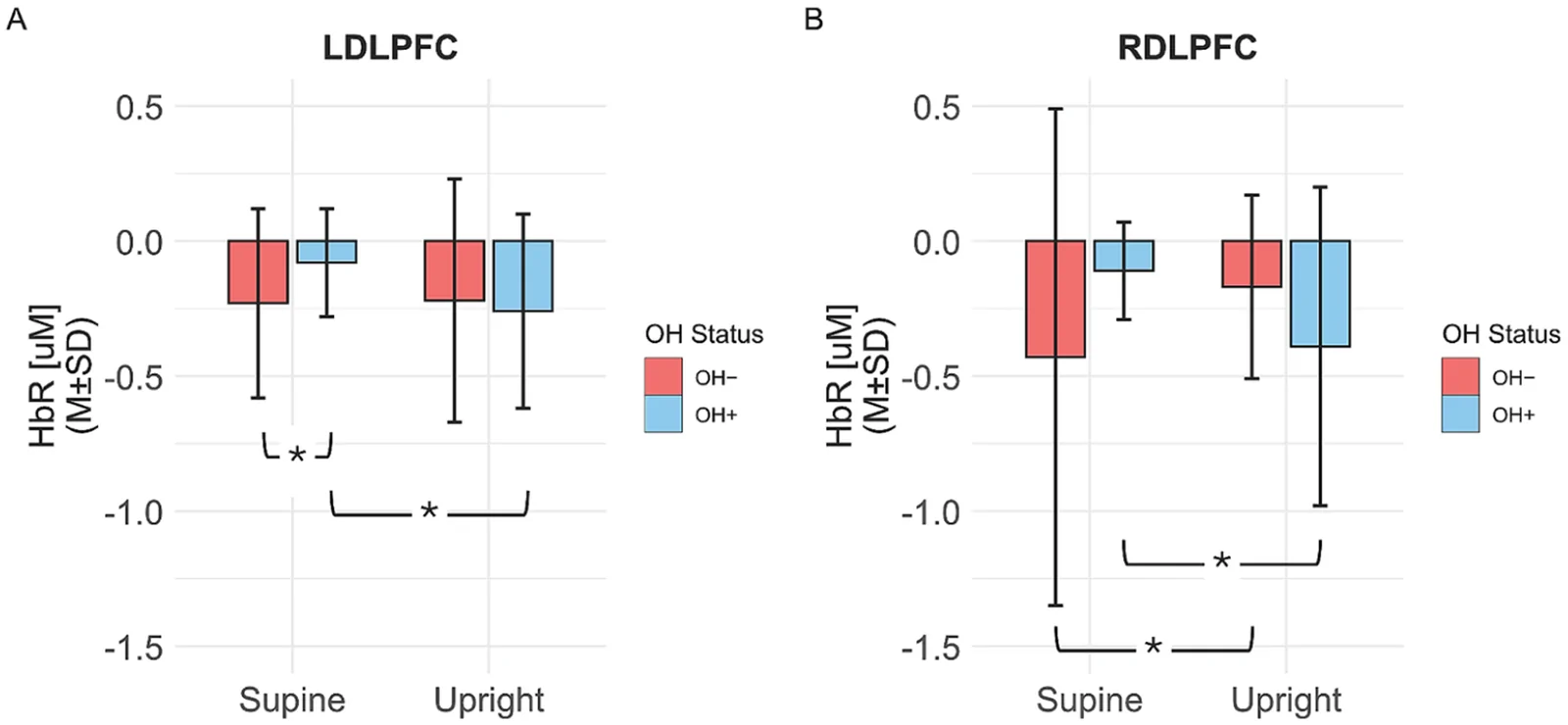
Deoxygenated hemoglobin (HbR) during the Delis-Kaplan Executive Function System letter verbal fluency test across the (A) left and (B) right dorsolateral prefrontal cortex (DLPFC) compared between Parkinson’s disease groups without orthostatic hypotension (OH−) and with orthostatic hypotension (OH+) and by position (supine vs. upright). Significance: * p < 0.05. Abbreviations: M, mean; SD, standard deviation; OH, orthostatic hypotension; OH−, without OH; OH+, with OH; HbR, deoxygenated hemoglobin; LDLPFC, left dorsolateral prefrontal cortex; RDLPFC, right dorsolateral prefrontal cortex.

The fNIRS data from four OH− participants were excluded due to poor signal quality. Descriptive statistics for fNIRS variables are summarized in Supplementary Table S2. Interactions between group and position were significant for HbR in both the left DLPFC (*χ*^2^(1) = 12.10, p < 0.001) and right DLPFC (*χ*^2^(2) = 11.52, *p* < 0.001). Post hoc results for the interaction between OH group and position are presented in Table 2. In the OH+ group, there was a significant decrease in HbR in both the left and right DLPFC in the upright position compared to the supine position, with moderate to large effect sizes. In the OH− group, there was a significant increase in HbR from supine to upright in the right DLPFC, with moderate to large effect size (Fig. 5). Post hoc comparisons of HbR between groups per position revealed a significant difference during the supine position, with the OH+ group having lower magnitude than the OH− group, with a moderate to large effect size. No significant differences were observed between groups during the upright position. Contrary to our hypothesis, there were no differences in HbO between groups or within groups across positions (Supplementary Table 3).

### 3.4. Orthostatic measurements during letter VFT

**Fig. 6.**
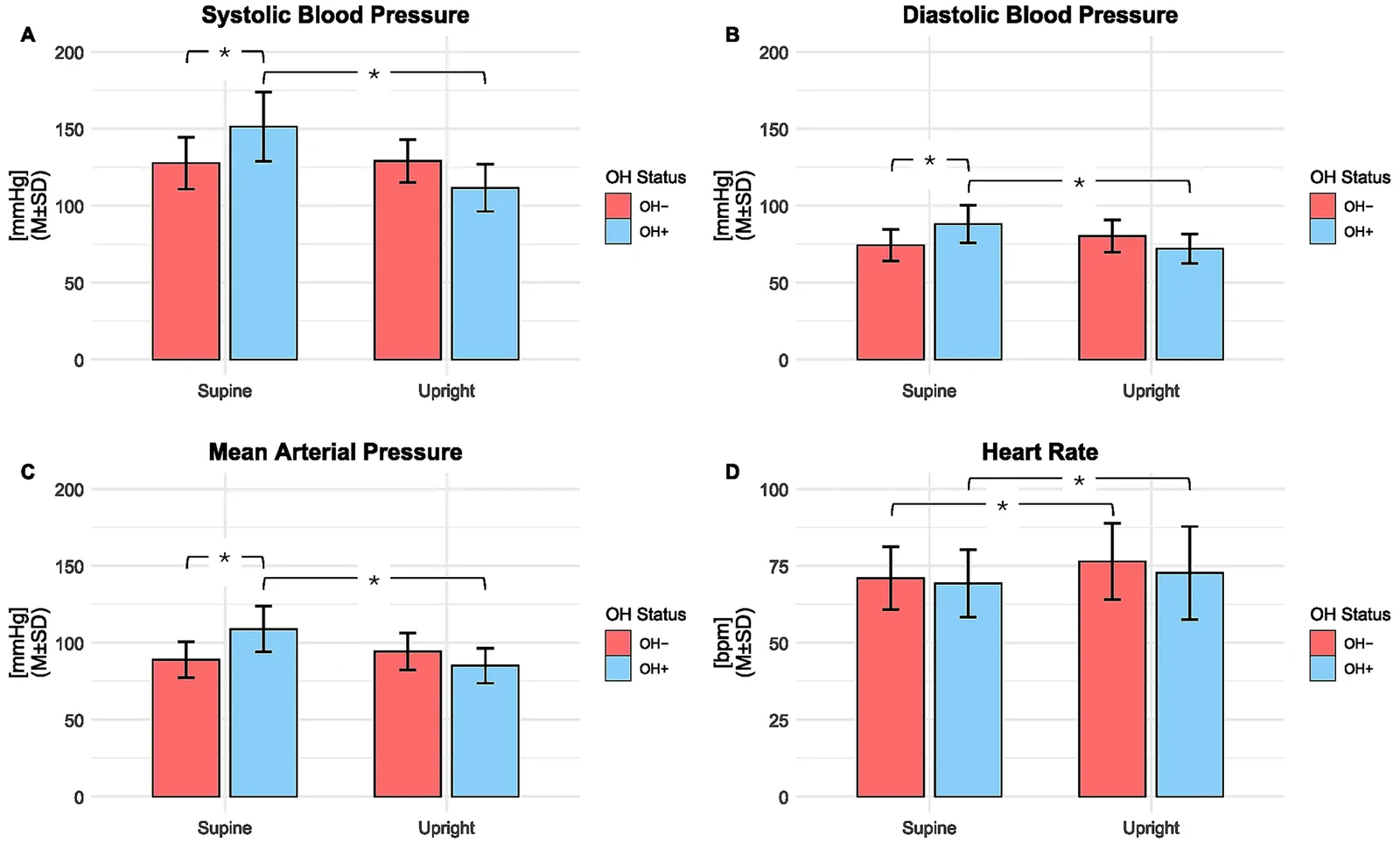
Between-group and within-group comparisons of orthostatic vital sign measurements during the Delis-Kaplan Executive Function System letter verbal fluency test: (A) systolic blood pressure; (B) diastolic blood pressure; (C) mean arterial pressure; (D) heart rate. Significance: * p < 0.05. Abbreviations: M, mean; SD, standard deviation; OH, orthostatic hypotension; OH−, without OH; OH+, with OH; mmHg, millimeters mercury; bpm, beats per minute.

Orthostatic vital sign measurements during performance of the letter VFT are shown in Fig. 6 and Supplementary Tables S4 and S5. The OH+ group had higher BP values than the OH− group in the supine position, which may reflect cardiovascular autonomic dysfunction and/or antihypotensive medication effects (DBP: t(35.0) = −2.78, *p* = 0.02, *d* = −1.29, 95% CI [−2.20, − 0.38]; MAP: t(35.0) = −3.48, *p* = 0.003, *d* =−1.64, 95% CI [−2.57, − 0.72]; SBP: t(34.9) = −3.01, *p* = 0.01, *d* = −1.46, 95% CI [−2.41, −0.51]) but not in the upright position (although upright SBP was marginally lower in the OH+ group than the OH− group (t(35.0) = 2.00, *p* = 0.05), *d* = 1.10, 95% CI [0.02, 2.18]). No between-group differences in HR were observed in the supine or upright position. As expected, the OH+ group had significantly lower BPs in the upright position than in the supine position (DBP: t(35.0) = 2.56, *p* = 0.03, *d* = 1.50, 95% CI [0.35, 2.64]; MAP: t(19.0) = 3.37, *p* = 0.01, *d* = 1.98, 95% CI [0.81, 3.15]; SBP: t(19.2) = 4.22, *p* < 0.001, *d* = 2.49, 95% CI [1.29, 3.69]). No postural differences in BP were observed in the OH− group. Both groups had a similar increase in HR when upright compared to supine (OH+: t(14.7) = −2.40, *p* = 0.03, *d* = −1.50, 95% CI [−4.66, 1.65]; OH−: t(14.9) = −3.57, *p* = 0.01, *d* = −1.50, 95% CI [−3.59, 0.58]). None of the participants reported orthostatic symptoms (e.g., lightheadedness, dizziness, or visual disturbances) during the upright letter VFT.

Sensitivity analyses: The physiologic OH group × position interaction estimates were similar in the primary all-available-data models and in complete-case analyses. The direction of the interaction was un-changed for SBP, DBP, MAP, and HR, and differences in effect magnitude were small. Complete-case models produced somewhat larger standard errors, consistent with the reduced number of observations, but did not materially alter the conclusions.

Covariate-adjusted sensitivity analyses also yielded interaction estimates that were directionally and quantitatively similar to the corresponding unadjusted estimates. Adjustment individually for age, sex, LEDD, and antihypotensive medication use did not materially change the OH group × position interaction for SBP, DBP, MAP, or HR. Results from the exploratory fully adjusted models were likewise consistent with the primary models. These findings suggest that the observed physiologic interaction effects were not substantially explained by the evaluated covariates, although residual confounding cannot be excluded because of the modest sample size. Detailed estimates of the sensitivity analyses are provided as Supplementary Tables S6–S8.

## 4. Discussion

This prospective observational study using fNIRS to examine positional cerebral oxygenation changes in participants with PD demonstrates that the effect of postural change on executive function (verbal letter fluency) performance differs by OH status. Although there were no significant main effects of group or posture in isolation, a robust Group × Position interaction emerged for letter fluency with a large effect size (partial η^2^ ≈ 0.15). OH− participants showed stable performance across positions (slightly enhanced in the upright position), whereas OH+ participants exhibited a relative decline in verbal letter fluency when upright compared to when supine. This pattern indicates that cognitive performance is not uniformly reduced in PD participants with OH, but is selectively vulnerable under orthostatic challenge. Although the Group × Position interaction for total errors did not reach statistical significance, the effect size was moderate, and the descriptive pattern paralleled that of letter fluency: OH+ participants tended to make more errors when upright than when supine, whereas OH− participants showed a slight reduction in errors when upright.

### 4.1. Cerebral hemodynamic changes during positional cognitive testing

Accompanying these position-dependent behavioral changes, the OH+ group exhibited a reduction in HbR during the letter fluency task when upright compared to supine, indicating greater cognitive resource demand in the upright position. In addition, the OH+ group showed lower engagement when supine relative to the OH− group, potentially reflecting greater demand being placed on the OH+ group. This finding raises the possibility that cerebral autoregulatory impairments in persons with PD and OH may not be confined to the upright posture and may reflect a broader vulnerability. Prior work has suggested that OH in PD is associated with impaired dynamic cerebral autoregulation while supine (Xing et al. 2022), potentially predisposing individuals to inefficient neurovascular responses during cognitive demands. Taken together, these findings suggest that the OH+ group may experience cerebral hemodynamic impairment in both positions, with greater impairment during the upright condition.

Contrary to our hypothesis, these position-related trends and changes were not observed in HbO. The discrepancy between a reduction in HbR when upright and the absence of corresponding HbO changes may suggest impaired neurovascular coupling, the physiological mechanism linking neural activity to local blood flow. Such decoupling has been observed in individuals with other causes of cardiovascular autonomic failure (Azevedo et al. 2011; Phillips et al. 2014) and may reflect altered neurogenic regulation and/or OH-related hypoperfusion that limits the expected hyperemic response (Phillips et al. 2016). Another interpretation is that HbR carries a stronger neurovascular coupling signal than HbO, especially when stimuli being employed are a composite of neuronal (VFT) and systemic (postural changes) components. Furthermore, systemic factors (cardiac oscillations and Mayer waves, which are related partially to BP) have a stronger effect on HbO signals and may obscure or mask the effects of task-related neuronal signals (Reddy et al., 2021).

Our work expands the limited body of literature characterizing posture-related cerebral oxygenation changes in individuals with cardiovascular autonomic failure. Prior work combining NIRS and TCD demonstrated greater posture-related reductions in HbO and mean cerebral blood velocity in participants with sympathetic failure compared with controls (Harms et al. 2000). Using diffuse optical tomography during dynamic and static head-up tilt, a subsequent study demonstrated progressive decreases in HbO and total hemoglobin that persisted during sustained tilt in participants with PD and OH; a more negative rate of HbO change was associated with greater autonomic symptom severity and the direction of HbO change distinguished participants with PD and OH from healthy controls (Kim et al. 2020). A multimodal NIRS-TCD study evaluated cerebral hemodynamic changes during head-up tilt and squat-to-stand transitions in PD participants with and without OH and healthy controls; PD participants with OH showed larger persistent left hemispheric HbO changes during head-up tilt and delayed peak times in the time derivative of total hemoglobin in the left hemisphere during squat-to-stand. In contrast, TCD-derived changes in mean flow velocity during head-up tilt did not significantly distinguish PD participants with and without OH (Joo et al. 2024). Taken together, these findings support abnormal posture-dependent cerebral oxygenation responses in OH+ individuals. However, these previous experimental paradigms did not address how hemodynamic abnormalities relate to task-evoked neural activation. The present study extends this literature by providing complementary evidence that position-dependent cerebral hemodynamic dysregulation in individuals with PD and OH may be associated with orthostatic impairments in verbal letter fluency.

### 4.2. Implications for clinical practice

Notably, the OH+ group demonstrated positional impairments in cognitive performance and cerebral oxygenation despite the absence of perceived orthostatic symptoms during testing. This dissociation has clinical implications, since the decision to initiate treatment for OH in PD typically relies on patient-reported symptoms such as dizziness or lightheadedness (Gibbons et al. 2017). However, persons with PD may exhibit hypotension unawareness, and lack typical warning signs of cerebral hypoperfusion (e.g., feeling faint) even during substantial BP reductions (Arbogast et al. 2009; Freeman et al. 2020). Our results suggest that reliance on symptom-based assessments alone may underestimate the extent of OH-related cerebral hypoperfusion, highlighting the value of fNIRS-derived measures for monitoring cerebral hemodynamics in patients with autonomic failure. In this context, fNIRS may offer a promising approach for more objective and physiologically relevant assessment of cerebral hemodynamics. By capturing real-time changes in cortical hemodynamics during orthostatic stress, fNIRS may identify patients with clinically meaningful hypoperfusion who would otherwise go unrecognized based on the lack of self-reported symptoms. This has potential implications for refining risk stratification, guiding earlier individualized interventions (e.g., behavioral or pharmacologic therapies for OH), and monitoring treatment response over time. fNIRS could also be implemented in clinical settings alongside autonomic testing – analogous to approaches used with TCD (Norcliffe-Kaufmann et al. 2018) – or integrated with neuropsychological assessments to provide a more comprehensive evaluation of brain function under orthostatic challenge. With further development, ambulatory fNIRS applications may also be feasible, enabling longitudinal monitoring in real-world environments. More broadly, incorporation of fNIRS-based biomarkers into clinical care could shift assessment from a symptom-driven to a physiology-informed paradigm, particularly in populations with impaired symptom awareness (Longardner et al., 2025b).

### 4.3. Influence of clinical and physiological covariates

We identified several between-group clinical differences that warrant further discussion. As expected, both between-group and within-group postural differences emerged in orthostatic BP measurements. The OH+ group had substantial BP reductions when transitioning from supine to upright despite continuation of routine antihypotensive medications, although these agents likely attenuated the magnitude of these reductions. In addition, the OH+ group took higher doses of levodopa than the OH− group, which is relevant given the known hypotensive effects of dopaminergic therapy (Bouhaddi et al., 2004; Cani et al., 2024; Noack et al., 2014; Longardner et al., 2025a). Although upright BP values were similar between groups, the OH+ group exhibited substantially higher supine BP values than the OH− group, reaching thresholds consistent with supine hypertension, defined as SBP ≥ 150 mmHg and/or DBP ≥ 90 mmHg (Gibbons et al. 2017). This is a common comorbidity among individuals with neurogenic OH (Fanciulli et al. 2016) and may also contribute to impaired cerebral autoregulation (Indelicato et al. 2015). We also observed a significant between-group difference in sex distribution, with males comprising most of the OH+ group. This likely reflects sampling bias (several participants were recruited from a local women’s PD support group and included in the OH− group) rather than a true disease-related pattern since prior studies have found no sex differences in the prevalence of OH in PD (Wang et al. 2025). Sex-specific cognitive differences have been described in PD, with males performing worse on global cognition, verbal fluency, and measures of inhibition and processing speed (Oltra et al. 2021; Reekes et al. 2020); however, our cohort did not show baseline differences in global cognition or letter fluency when supine. The absence of baseline disparities suggests that the observed decline in letter VFT performance when upright (relative to supine) is unlikely to be driven by sex and is instead a direct effect of OH.

### 4.4. Strengths and limitations

Strengths of our study include the use of non-invasive optical neuroimaging to assess posture-related changes in cognitive performance and cerebral hemodynamics in a well-characterized PD cohort. Several limitations should also be noted. The sample size was modest and unbalanced, particularly in the OH+ group, resulting in reduced precision of effect estimates and limited power to detect secondary outcomes. Enrollment began during the COVID-19 pandemic, which reduced recruitment during the first year, and the study visits were time-consuming and resource-intensive, which may have further limited participation.

Although age was included as a covariate and additional clinical variables were examined descriptively and, where feasible, in sensitivity analyses, the study was not powered to support simultaneous multivariable adjustment for several potentially relevant covariates. Residual confounding by age, sex, LEDD, and antihypotensive medication exposure therefore cannot be excluded, and the findings should be interpreted as preliminary and hypothesis-generating. Physiologic analyses were further limited by missing data and small sample sizes, particularly in the OH+ upright condition. Although mixed-effects models allow the use of all available data without listwise deletion, estimates from cells with few observations are less precise. These physiologic results should therefore be interpreted cautiously and viewed as complementary to the primary cognitive and fNIRS findings. Sensitivity analyses supported the robustness of the physiologic findings to the use of complete-case rather than all-available-data models and to adjustment for age, sex, LEDD, and antihypotensive medication use. However, these analyses do not eliminate the possibility of residual confounding. In addition, complete-case estimates were less precise due to a reduced sample size, particularly in the OH+ upright condition. The adjusted analyses should therefore be interpreted as supportive robustness checks rather than definitive multivariable estimates. In addition, participants continued their routine antiparkinsonian and antihypotensive medications during the study, which introduces potential confounding since these agents can influence BP, cerebral hemodynamics, and cognitive performance. Data regarding the timing of the last levodopa dose were unavailable for some participants, primarily in the OH− group. Among participants with available data, verbal fluency testing was completed approximately three hours after the last levodopa dose on average, beyond the expected peak acute antihypotensive effects (Longardner et al., 2025a). Therefore, while levodopa timing cannot be fully excluded as a contributor, it is unlikely to account for the observed posture-dependent cognitive and hemodynamic differences.

Another potential limitation is that we focused solely on letter verbal fluency as the primary cognitive measure. While the letter VFT is highly sensitive to PD-related impairment (Jacobs et al. 1995), the use of a single cognitive task limits our ability to determine whether similar patterns occur across a broader array of cognitive processes. It should be noted, however, that posture-dependent cognitive deficits affecting multiple domains have been previously demonstrated in OH+ individuals (Poda et al. 2012; Centi et al. 2017; Sforza et al. 2018). Similarly, our fNIRS analyses were limited to the DLPFC and did not evaluate other brain regions, which should be examined in future work (e.g., the occipital cortex during visuospatial tasks). In addition, temporal features of the hemodynamic response (e.g., peak latency) were not analyzed and may provide added insight into neurovascular coupling deficits. While our fNIRS system provides data about relative cerebral oxygenation trends, it cannot quantify absolute HbO/HbR values or cerebral blood flow velocity. Additional methods to measure cerebral hemodynamics, such as time-domain or frequency-domain fNIRS, diffuse correlation spectroscopy, or TCD, may further elucidate underlying physiological mechanisms. Finally, BP was measured intermittently using an oscillometric monitor, so we could not capture beat-to-beat BP fluctuations or precisely align systemic BP changes with fNIRS signals. Continuous BP monitoring would allow more accurate characterization of rapid hemodynamic shifts and their temporal relationship to cerebral oxygenation. Cognitive stress can transiently increase tremor and levodopa-induced dyskinesias in PD (Dirkx et al. 2020), which at times interfered with oscillometric BP acquisition during testing (although participants with large-amplitude involuntary movements were excluded). Since we did not directly measure cerebral perfusion or BP continuously during task performance, the inferred associations between orthostatic hemodynamic changes and fluency deficits remain indirect.

## 5. Conclusions and future directions

The current results provide converging evidence that OH interacts with posture to influence executive aspects of language production in PD. The selective impairment of letter fluency in OH+ participants when upright underscores the need to assess cognition under conditions that approximate the hemodynamic demands of everyday life and indicates a potentially important mechanism by which cardiovascular autonomic dysfunction contributes to functional cognitive complaints. Clarifying the mechanisms linking OH and cognitive impairment in PD is crucial, as recurrent OH-related cerebral hypoperfusion may contribute directly to both short- and long-term cognitive decline. If these relationships are causal, then effectively treating OH could help improve, prevent, or delay cognitive deterioration. Future work incorporating larger samples, a broader cognitive battery, and continuous BP monitoring should more clearly define the relationship between fNIRS-derived measures and systemic BP changes and identify the hemodynamic biomarkers that best predict and monitor postural impairments in cognitive function and cerebral oxygenation.

## Supplementary Material

supp1

supp3

supp2

## Figures and Tables

**Table 1:** Demographic, Clinical Characteristics, and Orthostatic Vital Sign Measurements of Enrolled Participants.

	OH− (n = 19)	OH+ (n = 10)
Sex, female	13 (68.4%)	**1 (10.0%)** **
Age, years	64.9 (6.1)	**70.8 (4.5)** **
Race	17 (89.5%)	9 (90.0%)
White/Caucasian	0 (0.0%)	1 (10.0%)
Asian	2 (10.5%)	0 (0.0%)
Other		
Ethnicity	17 (89.5%)	10 (100.0%)
Non-Hispanic/ Latino	2 (10.5%)	0 (0.0%)
Hispanic/Latino		
Education, years	17.3 (2.7)	18.7 (1.9)
PD duration, years	5.6 (3.2)	8.8 (6.2)
MDS-UPDRS Part III score (“on” state)	24.95 (12.1)	21.70 (11.7)
H&Y stage	1.8 (0.4)	1.8 (0.4)
LEDD, mg	394.2 (208.6)	**744.5 (416.5)** *
Time between last levodopa dose and VFT (minutes)	181.5 (90.1) (n = 10 missing)	193.0 (72.4) (n = 2 missing)
Taking antihypotensive medications	1 (5.3%)	**8 (80.0%)** ***
DRS-2 score	139.6 (3.6)	138.5 (3.0)
OHQ item 1	0.9 (2.0)	**3.1 (2.8)** *
OHSA composite score	1.0 (1.5)	2.2 (2.0)
Orthostatic Vital Sign Measurements During Screening Tilt		
SBP supine, mmHg	125.6 (17.4)	**159.8 (29.2)** ***
DBP supine, mmHg	69.6 (6.0)	**85.0 (16.1)** *
MAP supine, mmHg	88.3 (9.5)	**109.9 (19.3)** ***
HR supine, bpm	64.7 (10.4)	61.2 (6.8)
SBP upright, mmHg	123.8 (18.3)	118.2 (15.8)
DBP upright, mmHg	71.5 (9.9)	73.8 (11.2)
MAP upright, mmHg	89.0 (11.8)	88.6 (11.9)
HR upright, bpm	71.4 (11.9)	67.7 (10.2)
Δ SBP, mmHg	−1.8 (11.3)	**− 41.6 (19.10)**
Δ DBP, mmHg	+ 1.9 (6.1)	***
Δ MAP, mmHg	+0.7 (7.0)	**−12.6 (8.9)** ***
Δ HR, bpm	+7.6 (4.8)	**−21.3 (11.5)**
		***
		+6.4 (6.3)

Continuous variables are shown as mean (SD); categorical variables are shown as N (%).

Between-group comparisons were performed using independent t-tests for continuous variables and chi-squared test for categorical variables.

* p *<* 0.05,

** p *<* 0.01,

*** p *<* 0.001.

Abbreviations: DBP: diastolic blood pressure; DRS-2: Mattis Dementia Rating Scale-2; HR: heart rate; H&Y: Hoehn and Yahr; LEDD: levodopa equivalent daily dose; MAP: mean arterial pressure; OH−: without orthostatic hypotension; OH+: with orthostatic hypotension; OHSA: Orthostatic Hypotension Symptom Assessment; OHQ: Orthostatic Hypotension Questionnaire; PD: Parkinson’s disease; SBP: systolic blood pressure; VFT: verbal fluency test.

**Table 2:** Post hoc comparisons of deoxygenated hemoglobin (HbR) for the interaction between orthostatic hypotension (OH) group and position measured in the dorsolateral prefrontal cortex (DLPFC).

Brain Region	Group/ Position	Comparison	p value	d, 95% CI
Left DLPFC	OH−	Supine – Upright	0.791	−0.05 [−0.46, 0.35]
	OH+	Supine – Upright	**0.010**	0.73 [0.19, 1.27]
	Supine	(OH−) – (OH+)	0.141	−0.63 [−1.49, 0.22]
	Upright	(OH−) – (OH+)	0.714	0.15 [−0.69, 0.99]
Right DLPFC	OH−	Supine – Upright	**0.004**	−0.63 [−1.04, −0.22]
	OH+	Supine – Upright	**0.011**	0.73 [0.19, 1.27]
	Supine	(OH−) – (OH+)	0.108	−0.83 [−1.85, 0.19]
	Upright	(OH−) – (OH+)	0.291	0.53 [−0.48, 1.54]

## Appendix A. Supplementary material

Supplementary data to this article can be found online at https://doi.org/10.1016/j.clinph.2026.2112351.
