# Supplementary material for: Orthostatic hypotension and acute cognitive dysfunction in Parkinson’s disease: insights from functional near-infrared spectroscopy
Source: Clin Neurophysiol. Author manuscript; Available in PMC 2026 Aug 29. (PMC13523144; doi:10.1016/j.clinph.2026.2112351)
Supplement: Supplementary file 1 [file NIHMS2203139-supplement-supp1.docx]

| Supplementary Table 1: Techniques for Assessing Cerebral Hemodynamic Changes | | | | | |
| --- | --- | --- | --- | --- | --- |
|  | **Hemodynamic parameter measured** | **Upright/**  **positional assessment feasible?** | **Personnel / training required** | **Advantages** | **Limitations** |
| **TCD** | Real-time mean cerebral blood flow velocity from large diameter cerebral arteries | Yes | Trained operator/ technician | Continuous monitoring  High temporal resolution  Well established for dynamic assessment of cerebral autoregulation | Operator dependent  Requires stable insonation and patient stillness  Susceptible to movement artifact  Does not directly assess microvascular/regional perfusion  Relies on certain assumptions (e.g., arterial diameter remaining relatively constant)  Limited acoustic windows in some individuals; does not directly |
| **NIRS,**  **Continous-wave**  **fNIRS** | Relative changes in superficial cortical tissue oxygenation, estimated from oxygenated and deoxygenated hemoglobin concentrations | Yes | Minimal to moderate | Wearable and portable; can be used during postural transitions and naturalistic tasks  High temporal resolution  Relatively accessible | Limited primarily to superficial cortical regions; cannot directly assess deep structures  Susceptible to extracerebral/scalp blood flow effects, although short-separation channels can help mitigate this  Provides relative rather than absolute perfusion measures |
| **Frequency-domain fNIRS** | Absolute and relative changes in tissue absorption and scattering, enabling direct measurements of oxygenated and deoxygenated hemoglobin concentration | Yes | Moderate training; requires familiarity with instrumentation and calibration | Provides absolute quantification of hemoglobin concentrations  Better depth sensitivity than continuous-wave NIRS. | More complex instrumentation and analysis  Higher cost than continuous-wave systems  Still limited to superficial cortical regions  Susceptible to motion and extracerebral signal contamination |
| **ASL MRI** | Quantitative analysis of regional cerebral perfusion patterns | No; supine only | MRI technologist and imaging analysis expertise | Quantitative perfusion measure  Whole-brain coverage  Can assess cortical and subcortical regions, including basal ganglia | Low temporal resolution  Low signal to noise ratio  Cannot assess upright posture or rapid orthostatic transitions  Susceptible to motion artifact |
| **BOLD MRI** | Relative changes in regional blood oxygenation associated with neuronal activity | No; supine only | MRI technologist and imaging analysis expertise | Whole-brain coverage  High spatial resolution; can assess cortical and subcortical activation patterns | Indirect measure of neuronal activity and cerebral hemodynamics  Limited temporal resolution relative to TCD/fNIRS  Cannot assess upright posture or rapid orthostatic transitions  Susceptible to motion and systemic physiological artifacts |
| Abbreviations: ASL: Arterial spin-labeling; BOLD: Blood Oxygenation Level-Dependent; fNIRS: functional near-infrared spectroscopy; NIRS: near-infrared spectroscopy; TCD: Transcranial doppler | | | | | |

| Supplementary Table 2: Descriptive statistics of oxygenated (HbO) and deoxygenated hemoglobin (HbR) values in orthostatic hypotension (OH) groups across positions during the letter verbal fluency task measured from the dorsolateral prefrontal cortex (DLPFC). | | | | | | |
| --- | --- | --- | --- | --- | --- | --- |
| fNIRS variables | Region of interest | OH group | Position | n | Mean (μM) | Standard deviation |
| HbO | Left DLPFC | OH- | Supine | 51 | 0.52 | 0.58 |
| HbO | Left DLPFC | OH+ | Upright | 48 | 0.37 | 0.75 |
| HbO | Left DLPFC | OH- | Supine | 27 | 0.30 | 0.35 |
| HbO | Left DLPFC | OH+ | Upright | 30 | 0.37 | 0.50 |
| HbO | Right DLPFC | OH- | Supine | 51 | 0.43 | 0.77 |
| HbO | Right DLPFC | OH+ | Upright | 48 | 0.52 | 0.51 |
| HbO | Right DLPFC | OH- | Supine | 27 | 0.30 | 0.39 |
| HbO | Right DLPFC | OH+ | Upright | 30 | 0.29 | 0.48 |
| HbR | Left DLPFC | OH- | Supine | 51 | -0.23 | 0.35 |
| HbR | Left DLPFC | OH+ | Upright | 48 | -0.22 | 0.45 |
| HbR | Left DLPFC | OH- | Supine | 27 | -0.08 | 0.20 |
| HbR | Left DLPFC | OH+ | Upright | 30 | -0.26 | 0.36 |
| HbR | Right DLPFC | OH- | Supine | 51 | -0.43 | 0.92 |
| HbR | Right DLPFC | OH+ | Upright | 48 | -0.17 | 0.34 |
| HbR | Right DLPFC | OH- | Supine | 27 | -0.11 | 0.18 |
| HbR | Right DLPFC | OH+ | Upright | 30 | -0.39 | 0.59 |

| Supplementary Table 3: Post-hoc comparisons of oxygenated hemoglobin (HbO) for the interaction between orthostatic hypotension OH) group and position measured in the dorsolateral prefrontal cortex (DLPFC). | | | | |
| --- | --- | --- | --- | --- |
| Brain Region | Group/Position | Comparison | p value | d, 95% CI |
| Left DLPFC | OH- | Supine - Upright | 0.161 | 0.31 [-0.12, 0.74] |
|  | OH+ | Supine - Upright | 0.936 | 0.02 [-0.55, 0.59] |
|  | Supine | (OH-) - (OH+) | 0.354 | 0.56 [-0.66, 1.77] |
|  | Upright | (OH-) - (OH+) | 0.649 | 0.27 [-0.93, 1.48] |
| Right DLPFC | OH- | Supine - Upright | 0.593 | -0.11 [-0.53, 0.30] |
|  | OH+ | Supine - Upright | 0.851 | 0.25 [-0.60, 1.10] |
|  | Supine | (OH-) - (OH+) | 0.556 | 0.42 [-0.41, 1.24] |
|  | Upright | (OH-) - (OH+) | 0.309 | 0.05 [-0.51, 0.62] |

| Supplementary Table 4: Descriptive statistics of orthostatic blood pressure and heart rate changes during positional letter verbal fluency testing | | | | | |
| --- | --- | --- | --- | --- | --- |
| Position | Variable | OH- | | OH+ | |
|  |  | M ± SD | n | M ± SD | n |
| Supine | SBP [mmHg] | 128 ± 17.0 | 14 | 151 ± 22.5 | 7 |
|  | DBP [mmHg] | 74.3 ± 10.3 | 14 | 88.0 ± 12.2 | 7 |
|  | MAP [mmHg] | 89.0 ± 11.7 | 14 | 109 ± 14.9 | 7 |
|  | HR [bpm] | 71.1 ± 10.2 | 14 | 69.3 ± 11.0 | 7 |
| Upright | SBP [mmHg] | 129 ± 14.0 | 13 | 112 ± 15.4 | 5 |
|  | DBP [mmHg] | 80.3 ± 10.4 | 13 | 72.1 ± 9.5 | 5 |
|  | MAP [mmHg] | 94.3 ± 11.9 | 13 | 85.1 ± 11.3 | 5 |
|  | HR [bpm] | 76.5 ± 12.4 | 13 | 72.7 ± 15.1 | 5 |
| Abbreviations: M: mean, SD: standard deviation, OH: orthostatic hypotension, OH-: without OH, OH+: with OH, SBP: systolic blood pressure, DBP: diastolic blood pressure, MAP: mean arterial pressure, HR: heart rate, mmHg, millimeters mercury, bpm: beats per minute. | | | | | |

| Supplementary Table 5: Comparisons of orthostatic blood pressure and heart rate changes during positional letter verbal fluency testing between and within orthostatic hypotension groups. | | | | | | | | | | | | |
| --- | --- | --- | --- | --- | --- | --- | --- | --- | --- | --- | --- | --- |
| Variable | Within-Group | | | | | | Between-Group | | | | | |
|  | OH-: S vs U | | | OH+: S vs U | | | S: OH- vs OH+ | | | U: OH- vs OH+ | | |
|  | df | t | d  [LL, UL] | df | t | d  [LL, UL] | df | t | d  [LL, UL] | df | t | d  [LL, UL] |
| SBP  [mmHg] | 19.7 | -0.18 | -0.07  [-0.86, 0.72] | 19.2 | **4.22*** | 2.49  [1.29, 3.69] | 34.9 | **-3.01*** | -1.46  [-2.41, -0.51] | 35.0 | 2.00 | 1.10  [0.02, 2.18] |
| DBP [mmHg] | 35.0 | -1.45 | -0.56  [-1.32, 0.19] | 35.0 | **2.56*** | 1.50  [0.35, 2.64] | 35.0 | **-2.78*** | -1.29  [-2.20, -0.38] | 35.0 | 1.46 | 0.77  [-0.26, 1.80] |
| MAP [mmHg] | 19.4 | -1.11 | -0.43  [-1.20, 0.34] | 19.0 | **3.37*** | 1.98  [0.81, 3.15] | 35.0 | **-3.48*** | -1.64  [-2.57, -0.72] | 35.0 | 1.42 | 0.77  [-0.29, 1.82] |
| HR [bpm] | 14.9 | **-3.57*** | -1.50  [-3.59, 0.58] | 14.7 | **-2.40*** | -1.50  [-4.66, 1.65] | 23.3 | 0.39 | 0.52  [-2.11, 3.16] | 25.8 | 0.38 | 0.52  [-2.20, 3.24] |
| Abbreviations: S: supine, U: upright, OH: orthostatic hypotension, OH-: without OH, OH+: with OH, df: degrees of freedom, t: t-statistic for specified contrast, d: Cohen's d point estimate, LL: lower limit of 95% CI of Cohen's d, UL: upper limit of 95% CI of Cohen's d, SBP: systolic blood pressure, DBP: diastolic blood pressure, MAP: mean arterial pressure, HR: heart rate, mmHg, millimeters mercury, bpm: beats per minute. Significance: * p < 0.05. | | | | | | | | | | | | |

| Table 6: Missing data sensitivity analysis results comparing models fit to data with missing data and those fit to only data with compete cases. | | | | | | | | | | |
| --- | --- | --- | --- | --- | --- | --- | --- | --- | --- | --- |
| Outcome | All Data | | | Complete Case | | | Relative Change | SE Ratio | Same Direction | Robust to Complete Case |
|  | Estimate | SE | p | Estimate | SE | p |  |  |  |  |
| SBP | -34.86 | 5.10 | **0.000** | -36.84 | 6.33 | **0.000** | 0.06 | 1.24 | TRUE | TRUE |
| DBP | -16.74 | 3.20 | **0.000** | -16.85 | 4.20 | **0.000** | 0.01 | 1.31 | TRUE | TRUE |
| MAP | -22.97 | 3.55 | **0.000** | -24.19 | 4.62 | **0.000** | 0.05 | 1.30 | TRUE | TRUE |
| HR | 1.91 | 1.85 | 0.306 | 1.63 | 2.07 | 0.437 | 0.15 | 1.12 | TRUE | TRUE |
| Abbreviations: DBP: Diastolic blood pressure; HR: Heart rate; MAP: Mean arterial pressure; SBP: Systolic blood pressure | | | | | | | | | | |

| Table 7: Covariate sensitivity analysis results comparing primary model to model with the given covariate as an additional fixed effect. | | | | | | | | | | |
| --- | --- | --- | --- | --- | --- | --- | --- | --- | --- | --- |
| Covariate | Base Model | | Adjusted Model | | Relative Change | SE Ratio | Same Direction | CI Overlap | Robust | N |
|  | Estimate | SE | Estimate | SE |  |  |  |  |  |  |
| SBP |  |  |  |  |  |  |  |  |  |  |
| Antihypotensive medication | -34.86 | 5.10 | -34.85 | 5.10 | 0.000 | 1.00 | TRUE | TRUE | TRUE | 23 |
| LEDD | -34.86 | 5.10 | -34.92 | 5.11 | 0.002 | 1.00 | TRUE | TRUE | TRUE | 23 |
| Sex | -34.86 | 5.10 | -34.80 | 5.10 | 0.002 | 1.00 | TRUE | TRUE | TRUE | 23 |
| Age_c | -34.86 | 5.10 | -34.86 | 5.11 | 0.000 | 1.00 | TRUE | TRUE | TRUE | 23 |
| DBP |  |  |  |  |  |  |  |  |  |  |
| Antihypotensive medication | -16.74 | 3.20 | -16.73 | 3.20 | 0.001 | 1.00 | TRUE | TRUE | TRUE | 23 |
| LEDD | -16.74 | 3.20 | -16.74 | 3.20 | 0.000 | 1.00 | TRUE | TRUE | TRUE | 23 |
| Sex | -16.74 | 3.20 | -16.74 | 3.20 | 0.000 | 1.00 | TRUE | TRUE | TRUE | 23 |
| Age_c | -16.74 | 3.20 | -16.75 | 3.20 | 0.001 | 1.00 | TRUE | TRUE | TRUE | 23 |
| MAP |  |  |  |  |  |  |  |  |  |  |
| Antihypotensive medication | -22.97 | 3.55 | -22.95 | 3.55 | 0.001 | 1.00 | TRUE | TRUE | TRUE | 23 |
| LEDD | -22.97 | 3.55 | -22.97 | 3.56 | 0.000 | 1.00 | TRUE | TRUE | TRUE | 23 |
| Sex | -22.97 | 3.55 | -23.00 | 3.56 | 0.002 | 1.00 | TRUE | TRUE | TRUE | 23 |
| Age_c | -22.97 | 3.55 | -22.97 | 3.55 | 0.000 | 1.00 | TRUE | TRUE | TRUE | 23 |
| HR |  |  |  |  |  |  |  |  |  |  |
| Antihypotensive medication | 1.91 | 1.85 | 1.91 | 1.85 | 0.001 | 1.00 | TRUE | TRUE | TRUE | 23 |
| LEDD | 1.91 | 1.85 | 1.91 | 1.85 | 0.000 | 1.00 | TRUE | TRUE | TRUE | 23 |
| Sex | 1.91 | 1.85 | 1.90 | 1.85 | 0.001 | 1.00 | TRUE | TRUE | TRUE | 23 |
| Age_c | 1.91 | 1.85 | 1.91 | 1.85 | 0.000 | 1.00 | TRUE | TRUE | TRUE | 23 |
| Abbreviations: DBP: Diastolic blood pressure; HR: Heart rate; LEDD: Levodopa equivalent daily dose MAP: Mean arterial pressure; SBP: Systolic blood pressure | | | | | | | | | | |

| Table 8: Exploratory covariate sensitivity analysis results comparing primary model to model with all given covariates as additional fixed effects. | | | | | | | | | | | | |
| --- | --- | --- | --- | --- | --- | --- | --- | --- | --- | --- | --- | --- |
| Outcome | Base Model | | | Adjusted Model | | | Relative Change | SE Ratio | Same Direction | CI Overlap | Robust | N |
|  | Estimate | SE | p | Estimate | SE | p |  |  |  |  |  |  |
| SBP | -34.86 | 5.10 | 0.00 | -34.76 | 5.11 | 0.00 | 0.003 | 1.00 | TRUE | TRUE | TRUE | 23 |
| DBP | -16.74 | 3.20 | 0.00 | -16.69 | 3.20 | 0.00 | 0.003 | 1.00 | TRUE | TRUE | TRUE | 23 |
| MAP | -22.97 | 3.55 | 0.00 | -22.88 | 3.56 | 0.00 | 0.004 | 1.00 | TRUE | TRUE | TRUE | 23 |
| HR | 1.91 | 1.85 | 0.31 | 1.90 | 1.85 | 0.31 | 0.001 | 1.00 | TRUE | TRUE | TRUE | 23 |
| Abbreviations: DBP: Diastolic blood pressure; HR: Heart rate; MAP: Mean arterial pressure; SBP: Systolic blood pressure | | | | | | | | | | | | |
