# Supplementary material for: Orthostatic hypotension and acute cognitive dysfunction in Parkinson’s disease: insights from functional near-infrared spectroscopy
Source: Clin Neurophysiol. Author manuscript; Available in PMC 2026 Aug 29. (PMC13523144; doi:10.1016/j.clinph.2026.2112351)
Supplement: Supplementary file 2 [file NIHMS2203139-supplement-supp3.docx]

**Supplementary Material for Statistical Analyses**

The following report details the assumption tests conducted for the 2 × 2 Mixed Model ANCOVA.

**Assumption Testing Report**

Before interpreting the primary analysis of covariance (ANCOVA), examining the effects of orthostatic hypotension (OH) status and body position on cognitive performance, a comprehensive check of statistical assumptions was performed. The covariate was Age, and the dependent variables were Verbal Fluency Scores and Total Errors.

**1. Homogeneity of Regression Slopes**

This assumption checks whether the relationship between the covariate (Age) and the dependent variable is consistent across all experimental conditions. A significant interaction would indicate that the covariate behaves differently across groups or positions, violating the ANCOVA assumption.

- **Method:** A mixed model was fitted, including the interaction terms between Age and the independent variables (Group and Position).
- **Results:**
  - **Verbal Fluency:** No significant interactions were found between Age and Group (p=0.434), Age and Position (p=0.638), or the three-way interaction (p=0.525).
  - **Total Errors:** Similarly, there were no significant interactions involving Age (all p > 0.05).
- **Interpretation:** The assumption of homogeneity of regression slopes is met for both dependent variables. This confirms that Age is a valid covariate that can be adjusted for uniformly across all groups.

**2. Normality of Residuals**

This assumption requires that the errors (residuals) of the model are normally distributed.

- **Method:** The Shapiro-Wilk test was applied to the residuals of the final mixed models.
- **Results:**
  - **Verbal Fluency:** The test was non-significant (W = 0.980, p = 0.471), indicating normal distribution.
  - **Total Errors:** The test was significant (W = 0.950, p = 0.018), suggesting a deviation from normality.
- **Interpretation:** The assumption is met for Verbal Fluency. For Total Errors, the violation is expected, given that error counts are discrete and skewed (Poisson-like). While Mixed Models are robust to minor violations, the bootstrapped confidence intervals provided in the primary analysis (which do not assume normality) should be prioritized for inference regarding Errors.

**3. Homogeneity of Variance (Homoscedasticity)**

This assumption checks if the variability in scores is similar between the OH- and OH+ groups at each position.

- **Method:** Levene's Test for Equality of Variances was conducted for the Supine and Upright positions separately.
- **Results:**
  - **Verbal Fluency:** No significant differences in variance were found for Supine (W=0.03, p=0.85) or Upright (W=0.17, p=0.69) positions.
  - **Total Errors:** No significant differences were found for Supine (W=0.46, p=0.50) or Upright (W=0.62, p=0.44) positions.
- **Interpretation:** The assumption of homogeneity of variance is fully met for both outcome measures.

**4. Linearity**

This assumption requires a linear relationship between the covariate (Age) and the dependent variable.

- **Method:** Visual inspection of scatter plots (Age vs. Scores).
- **Results:**
  - **Verbal Fluency:** The plot shows a weak negative trend consistent with expected age-related decline, with no obvious curvilinear patterns.
  - **Total Errors:** The plot shows a weak positive trend (errors increasing slightly with age).
- **Interpretation:** The linear specification for the Age covariate is appropriate.

**Summary Table of Assumptions**

| **Assumption** | **Verbal Fluency** | **Total Errors** | **Action / Note** |
| --- | --- | --- | --- |
| **Homogeneity of Regression Slopes** | Met (p > .05 ) | Met ( p > .05 ) | Proceed with Age as a covariate. |
| **Normality of Residuals** | Met ( p = .47 ) | **Violated** ( p = .018 ) | Use Bootstrapping for Errors. |
| **Homogeneity of Variance** | Met ( p > .05 ) | Met ( p > .05 ) | No correction needed. |
| **Linearity** | Met (Visual) | Met (Visual) | The Linear Age term is valid. |

**Conclusion:** The data largely meet the assumptions for a Mixed Model ANCOVA. The primary concern is the non-normality of the Error data, which has been mitigated in the primary analysis by using robust cluster bootstrapping to generate confidence intervals.
